# Characterization of four functional biocompatible pressure-sensitive adhesives for rapid prototyping of cell-based lab-on-a-chip and organ-on-a-chip systems

**DOI:** 10.1038/s41598-019-45633-x

**Published:** 2019-06-26

**Authors:** S. R. A. Kratz, C. Eilenberger, P. Schuller, B. Bachmann, S. Spitz, P. Ertl, M. Rothbauer

**Affiliations:** 1 Institute of Applied Synthetic Chemistry and Institute of Chemical Technologies and Analytics, Faculty of Technical Chemistry, Vienna University of Technology, Vienna, Getreidemarkt 9/163-164, 1060 Vienna, Austria; 2 Austrian Cluster for Tissue Regeneration, Vienna, Austria; 3 Ludwig Boltzmann Institute for Experimental and Clinical Traumatology, Allgemeine Unfallversicherungsanstalt (AUVA) Research Centre, Donaueschingenstraße 13, 1200 Vienna, Austria

**Keywords:** Lab-on-a-chip, Apicobasal polarity

## Abstract

In the advent of affordable photo- and soft-lithography using polydimethylsiloxane (PDMS), low cost multi-step microfabrication methods have become available to a broad scientific community today. Although these methods are frequently applied for microfluidic prototype production in academic and industrial settings, fast design iterations and rapid prototyping within a few minutes with a high degree of flexibility are nearly impossible. To reduce microfluidic concept-to-chip time and costs, a number of alternative rapid prototyping techniques have recently been introduced including CNC micromachining, 3D printing and plotting out of numeric CAD designs as well as micro-structuring of thin PDMS sheets and pressure sensitive adhesives. Although micro-structuring of pressure sensitive adhesives promises high design flexibility, rapid fabrication and simple biochip assembly, most adhesives are toxic for living biological systems. Since an appropriate bio-interface and proper biology-material interaction is key for any cell chip and organ-on-a-chip system, only a limited number of medical-grade materials are available for microfluidic prototyping. In this study, we have characterized four functional biomedical-grade pressure sensitive adhesives for rapid prototyping (e.g. less than 1 hour) applications including structuring precision, physical and optical properties as well as biocompatibilities. While similar biocompatibility was found for all four adhesives, significant differences in cutting behavior, bonding strength to glass and polymers as well as gas permeability was observed. Practical applications included stability testing of multilayered, membrane-integrated organ-on-a-chip devices under standard cell culture conditions (e.g. 2–3 weeks at 37 °C and 100% humidity) and a shear-impact up to 5 dynes/cm^2^. Additionally, time- and shear-dependent uptake of non-toxic fluorescently labelled nanoparticles on human endothelial cells are demonstrated using micro-structured adhesive-bonded devices. Our results show that (a) both simple and complex microdevices can be designed, fabricated and tested in less than 1 hour, (b) these microdevices are stable for weeks even under physiological shear force conditions and (c) can be used to maintain cell monolayers as well as 3D cell culture systems.

## Introduction

The introduction of affordable microfabrication techniques and bench-top size equipment over a decade ago has obviated the dependence on costly cleanroom facilities for most microfluidic applications^1^. As a consequence interest in microfluidic devices and lab-on-a-chip technologies have steadily grown in recent years with highest impact in biology and biomedical research using customized organ-on-a-chip systems to study tissue responses and organotypic cell assemblies^2–6^. Here, the global trend towards more complex 3-dimensional biological structures that exhibit physiological relevant functionalities are accommodated by new microfabrication possibilities to generate micro physiological cell culture platforms. This means that cell chips and organ-on-a-chip system can (a) be fabricated using 2D and 3D geometries and complex microchannel network, (b) be based on a variety of biocompatible materials and (c) may contain integrated sensors and actuators, which are needed to perform cell culture handling, cell manipulation and analysis^7^. Despite these advantages and market opportunities, development costs of cell-based lab-on-a-chips and organ-on-a-chip systems are still high and the period needed to go from initial idea to functional prototypes to final product can take up to several years prior mass production. This lengthy development time can in part be attributed to an increased number of design iterations required to miniaturize cell-based assays and to integrate living biology into microdevices. It is important to note that standard cell culture techniques are optimized for static conditions using large cell numbers and high medium volumes employing coated polystyrene flasks and culture plates. This means that integration of living cell cultures into microfluidic devices and miniaturization of cell-based assays is not straight forward, does not follow simple scaling laws and in many cases requires an empirical approach to adjust oxygen demands, nutrient supply, waste removal and the application of adequate shear-force conditions. Consequently, rapid prototyping methods are key for cost and time reduction, since they offer fast design alterations resulting in improved cell culture optimization and feasibility studies.

**Figure 1:**
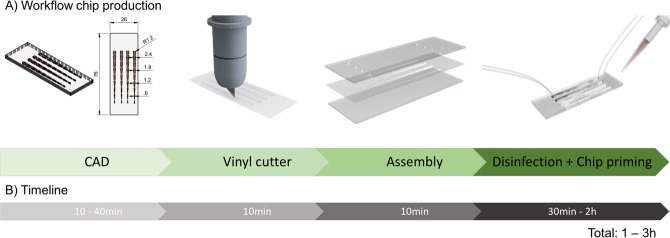
(**A**) Process flow and (**B**) time investment for rapid prototyping of pressure sensitive biomedical adhesives (PSAs).

While frequently employed microfabrication methods such as photolithography^8^, soft-lithography^9^, laser machining^10^, hot embossing^11^ and three-dimensional printing^12^ enable biochip designs with highest microscale resolution^13,14^, low-resolution devices are generally sufficient for cell culture applications. Although, a number of rapid, low-resolution and low-cost methods that may even allow the fabrication of microdevices on site^15,16^ have been developed for remote-access and point-of-care solutions, these devices are in many cases not amenable for cell culture applications. Since cell-based lab-on-a-chip and organ-on-a-chip systems need to reconstruct the micro physiological niche necessary to maintain the respective cell culture model *in vitro*^17–24^, the bio-interface governing the biology-material interaction becomes a determine factor in the design of cell chips. It is important to highlight that only an appropriate cell culture interface promotes physiological cellular behavior, which restricts available chip bonding and assembly methods to thermal and some chemical procedures in regards to their biocompatibility^25–27^. Reliable biochip bonding and assembly is particularly important when using multi-layered, membrane-integrated and multi-material-based biochips, since simple bonding strategies using glues tend to be highly toxic for cells. These multi-layered devices are, however, highly popular where a physical barriers is needed to inhibit cell migration while simultaneously allowing the exchange of soluble signaling molecules through the pores, thus mimicking the role of a tissue barriers *in vivo*. Consequently, multi-layered and membrane-integrated microdevices have been extensively applied to study locally separated tissue-to-tissue interactions, various immune responses, bimolecular transport characteristics, gas and fluid exchange behavior, drug delivery and uptake mechanisms as well as nanoparticle absorption^28,29^. A prominent microfabrication technique used to fabricate multi-layered cell-based lab-on-a-chip system sandwiches a porous membrane between polydimethylsiloxane (PDMS) sheets bonded to glass substrates. Although casting elastomeric PDMS can rigorously reduce the concept-to-chip time down to several days^30^ and reduce costs^31^, soft-lithography still requires CAD designing of a photomask for UV photolithography to generate the required master molds. While soft-lithography is inexpensive and easy to use, it comes however along with a number of drawbacks including vapor permeability that leads to bubble formations inside cultivation chambers and small hydrophobic molecule adsorption, which can significantly change apparent drug concentrations and concentration gradients inside cultivation chamber^32^. Additionally, PDMS may cause problems for on-chip cell cultivation and manipulation through the leaching of uncross linked oligomers, deformation of channels and cell chambers, as well as gas permeability, which can lead to evaporation and changes in medium composition and shifts in osmolarity^33^. Furthermore, PDMS devices are known (a) for their short-term stability of surface treatments, (b) malfunction under high-pressure operations, and (c) are incompatibility with industrial scale-up and mass production processes^34,35^. This means that scaling up biochip fabrication requires different manufacturing methods and materials to go from small to medium to large volume manufacturing of cell chips. It is important to highlight that each transition from soft-lithography using PDMS to hot-embossing of thermoplastic industrial polymers^36^ to injection-molding of pre-polymerized molten thermoplastic granules^36–38^ necessitates iterative development of a series of prototypes to ensure device quality, functionality and biocompatibility prior mass production (see also Fig. 1A).

To reduce the biochip development time and costs associated with the transition from academic to industrial changing manufacturing methods, rapid one-step prototyping technologies are needed to go from idea to functional prototype to final product^39^. As an example, a reduction in concept-to-chip time can be achieved by using pressure sensitive adhesive tapes that can been micro-structured and used to bond a large variety of industrial-relevant polymeric materials including polymethylmethacrylate (PMMA), polycarbonate (PC), cyclic olefin polymer (COC/COP), polystyrene (PS), and polyethylene terephthalate (PET/PETG) (Supplementary Fig. 1). In other words, similar polymeric material can now be used throughout the entire biochip development process while rapid design iteration and assembly is accomplished by micro-structuring thin flexible adhesive polymer film. Although a number of pressure-sensitive adhesive tapes exists, only a handful can be used in biomedical research and clinical settings. In this study we set out to characterize and compare four medical grade pressure-sensitive adhesives^40^, three acrylic and one silicone-adhesive-based pressure sensitive adhesive for prototyping precision, physical and optical properties such as bonding strength, vapor and gas transparency as well as biocompatibility. As practical applications, a multilayered, membrane-integrated cell chip and a single compartment microfluidic biochip are fabricated and tested for (a) its stability under prolonged cell culture conditions and (b) to investigate shear-dependent uptake of non-toxic fluorescently labelled nanoparticles on human endothelial cells.

## Results

### Rapid prototyping of cell-based biochips using medical-grade pressure-sensitive adhesive tapes

Initial characterization of the four biomedical grade pressure-sensitive adhesive tapes set out to investigate bonding strengths and microstructure resolution. Different material composition and thickness of the pressure sensitive adhesives based on the manufactures data sheet (Adhesive research) are shown in Table 1.

**Table 1:** Composition of biomedical grade tapes.

Name	Total thickness	Layer thickness	Adhesives thickness	Adhesives type
ARcare 92712®	48.26 μm	12.7 μm polyester	17.78 μm	MA-93 acrylic pressure sensitive
ARcare 90445^®^	81.28 μm	25.4 μm polyester	27.94 μm	AS-110 acrylic medical grade
ARcare 90106^®^	142.24 μm	25.4 μm polyester	58.42 μm	MA-69 acrylic hybrid medical grade
ARseal 90880^®^	142.24 μm	50.8 μm polypropylene	45.72 μm	SR-26 silicone adhesive

**Figure 2:**
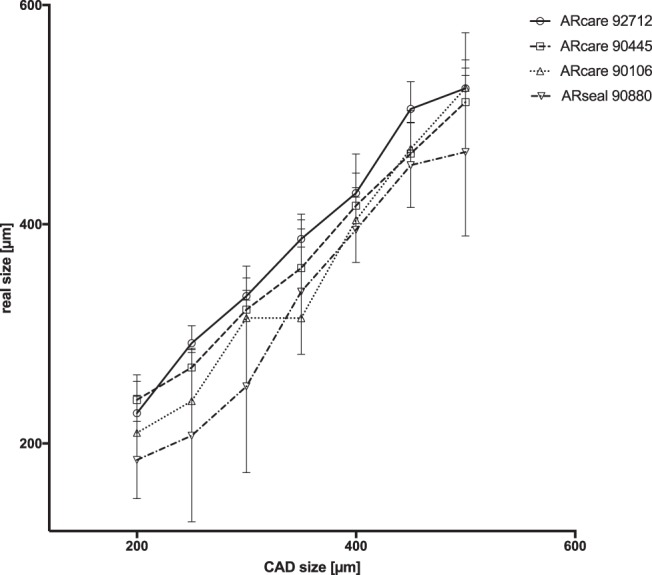
Comparison between numerical design and actual dimensions of microfluidic structures out of double-sided pressure sensitive biomedical adhesives after plotter rapid prototyping. Data points are presented as mean values ± SD for n = 3.

To evaluate the degree of three-dimensional precision that can be obtained using a vinyl plotter the same numerical CAD design was used to compare achieved microfluidic structures between the four adhesive tapes. Results of our precision study are shown in Fig. 2 indicating that the adhesive tape ARcare 90106 performed best when creating structures between 200–300 µm, whereas ARcare 90445 and ARcare 92712 exhibited increased structure sizes of 240 ± 23.51 µm and 228 ± 27,56 µm using 200 µm design features. In turn, adhesive tape ARseal 90880 displayed 20% smaller structures compared to the numerical model, which can be attributed to the high viscosity of the silicone adhesive layers. Compared to PDMS (SI Fig. 2) where the cutting resolution is limited by the device resolution, the adhesive layer of the PSAs are the main restriction to for the precision of the cutting process. Although structures above 300 µm to 500 µm in size showed similar precision, the adhesive tape ARseal 90880 exhibit highest RSD values around 18%, which can be linked to the 2 to 4-fold higher thickness of the 50.8 μm polypropylene film. Overall, ARcare 90106 performed best for structural resolution during rapid prototyping with RSDs around 11.5%.

**Figure 3:**
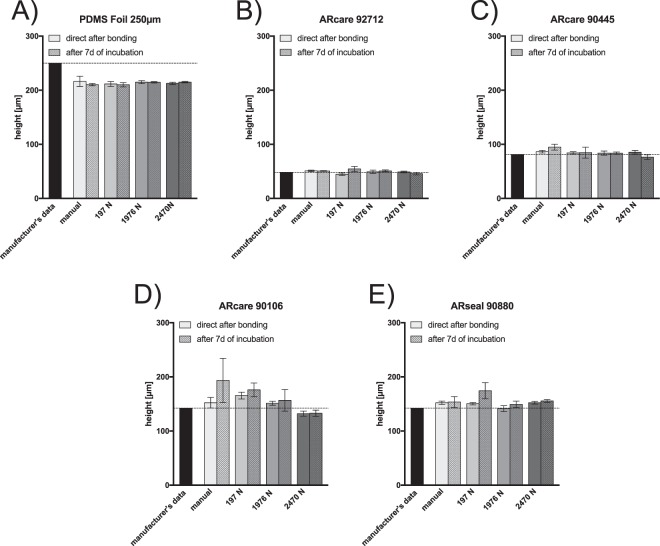
Comparison between manufacturer’s height specifications and actual microstructure height of (**A**) Super clear PDMS foil 250 µm (**B**) ARcare 92712, (**C**) ARcare 90445, (**D**) ARcare 90106 and (**E**) ARseal 90880 directly after bonding and after exposure to cell culture conditions for 7 days (100% humidity, 37 °C and 5% CO_2_). Data points are presented as mean values ± SD for n = 3.

Next the influence of applied bonding pressures on height variations and device stability during prolonged cell culture conditions was investigated to identify optimal assembly conditions of each of the four pressure-sensitive adhesive tapes. A comparison between manufacturer’s height specifications and actual microstructure thickness during an applied bonding pressure of 197 N equal to 10 N/cm^2^ yielded significant size variations for all ARseal adhesives tapes (see Fig. 3A–D). Additionally, manual bonding practice resulted in deviations from the manufacturer’s height specifications. Since chamber high variations, thus available medium volume, are known to affect cell culture conditions, additional swelling effects over a period of 7 days under humid cell culture conditions at 37 °C were investigated. Results shown in Fig. 3 indicate significant increases in microstructure height for all pressure-sensitive adhesive tapes (at 10 N/cm^2^), thus pointing at an inflation of the adhesive tapes over time at elevated temperatures and 100% humidity. Interestingly a 10-fold increase in bonding pressure to 1976 N/cm^2^ eliminated the swelling effect and resulted in microfluidic channels, thus pointing at the importance of defined bonding pressure profiles to obtain reproducibility, reliability and stable microstructure heights under cell culture conditions. Furthermore, an increase to 2470 N force for the bonding process lead to no differences in height for ARcare 92712, ARcare 90445 and ARseal 90880 as well as a decrease in height for ARcare 90106. However, the height of the PDMS was not influenced by the applied bonding pressure. Knowledge on the actual microchannel geometry is of particular relevance when investigating the effects of shear on cell physiology. In other words, employing incorrect channel heights leads to miscalculation of apparent fluid mechanical forces that act on the cell culture under investigation, thus resulting on misinterpretation of cellular behaviors.

### Characterization of multi-layered and membrane-integrated biochips assembled using pressure-sensitive adhesive tapes for cell culture applications

**Figure 4:**
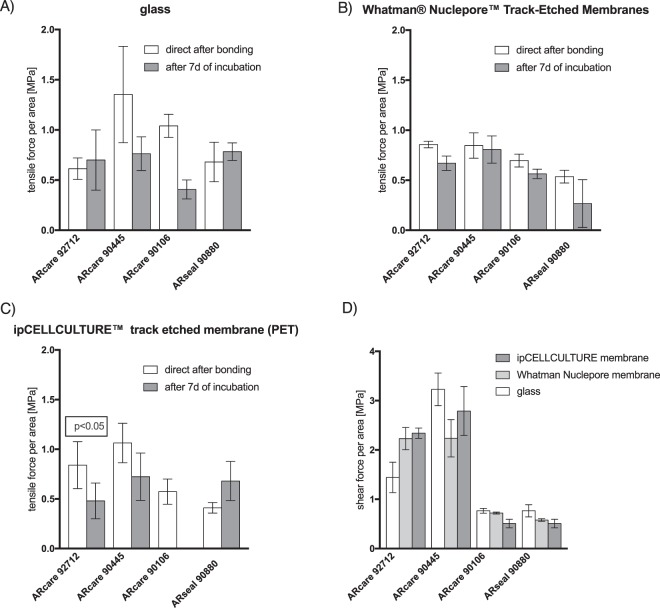
(**A**) Tensile force: glass-PSA-glass (**B**) tensile force: glass-PSA-membrane-PSA-glass (**C**) tensile force: glass-PSA-membrane-PSA-glass and (**D**) shear force-to-failure characterization of ARcare and ARseal pressure sensitive adhesives for glass and porous membrane bonding substrates. Data points are presented as mean values ± SD for n = 3.

Since cell-based lab-on-a-chip systems often contain functional components such as flexible membranes to biomechanically stimulate cell cultures or rigid porous membranes to allow for cell separation and polarization, the ability of adhesive tapes to readily assemble multi-material/hybrid devices is investigated in subsequent experiments. Results in Fig. 4A–D summarize obtained tensile force and shear force-to-failure directly after bonding (applied pressure of 2 kN/cm^2^) and after 7 days incubation at 37 °C and 100% humidity. Here, approx. 1 cm^2^ sheet of pressure sensitive adhesive tapes were layered between two glass slides containing various integrated porous membrane materials such as PC, PET, and PES. Results shown in Fig. 4A–C reveal that ARcare 90445 performed best for any material combination, while the other two ARcare adhesives showed a significant decrease of bonding strength towards polyester membranes between directly after bonding and after 7-days of incubation (ARcare 92712 with p < 0.05 by t-test) or even bonding-failure after the 7-day incubation period (e.g. ARcare 90106). Additionally, ARcare adhesive tapes demonstrated improved bonding strength with polycarbonate (e.g. track-etched Whatmann nuclepore membranes) for both bonding test scenarios over ARseal silicone-based adhesive types. Furthermore, 2 to 3-fold higher shear forces were necessary to delaminate ARcare 92712 and ARcare 90445 when compared to ARcare 90106 and ARseal adhesive tapes (see Fig. 4D). PDMS with a bonding strength to glass of 1.38 MPa (tensile) and 329.3 MPa (shear) (data not shown) shows quite a sufficient bonding ability to glass but has the drawback that they do not bond to polymer membrane without any further chemical treatment. These results confirm that pressure sensitive biomedical adhesives are an easy, robust alternative bonding strategy to assemble multiple layers of different polymer types with ARcare 90445 performing best among the tested biomedical-grade adhesives with respect to tensile and shear tests on glass and polymeric membranes.

Since material properties play a key role in microfluidic cell culture applications, oxygen and vapor permeability as well as optical transparency including autofluorescence were investigated in more detail in the next set of experiments. While transfer of oxygen permeability was monitored using integrated oxygen microsensors^20,24^, vapor permeability was measured indirectly via increase of air bubble volume over time.

**Figure 5:**
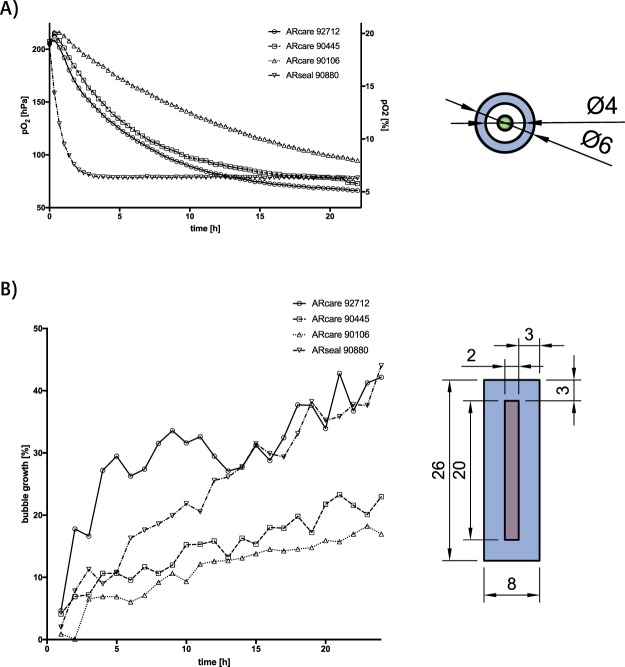
(**A**) Oxygen: where in a circular structure with a wall thickness of 1 mm (blue units in mm) oxygen is measured by micro sensor (green) and (**B**) vapor permeability of biomedical pressure sensitive adhesives: where medium (red) evaporation is measured through rectangular structure with a wall thickness of 3 mm (blue all units in mm) Data points are presented as mean values for n = 4. For mean values ± SD see SI Fig. 4.

Results shown in Fig. 5 clearly indicate that ARseal silicone-based adhesives, which contain two 45.72 μm of SR-26 silicone adhesive layers (see Table 1), exhibits highest oxygen and vapor permeability. This means that ARseal silicone-based adhesive essentially behaves like polydimethylsiloxane-based biochips reaching hypoxic conditions (e.g. defines as 5% oxygen level) already after 1.5 h. The increased gas permeability is also reflected in the observed vapor loss resulting in 40% microbubble growth within 24 hours. In turn, ARcare 90445 and ARcare 92712 adhesive tapes displayed lower gas exchange rates reaching 5% hypoxic condition only after 24 hours, which amounts to a 16-fold lower oxygen permeability. Interestingly, ARcare 92712 displayed a significant higher vapor permeability (40% bubble growth) compared to 20% for ARcare 90445 (p < 0.001), while in the presence of ARcare 90106 5% oxygen level was not reached within 24 hours and only 12% bubble growth was observed. Even though ARcare 90106 consists of the same 25.4 µm polyester film as ARcare 90445, its MA-69 acrylic hybrid medical adhesive, which takes up 82% of the total tape thickness, is less permeable to gas and vapor than the AS-110 acrylic adhesive layer of ARcare 90445 corresponding to 69% total thickness.

**Figure 6:**
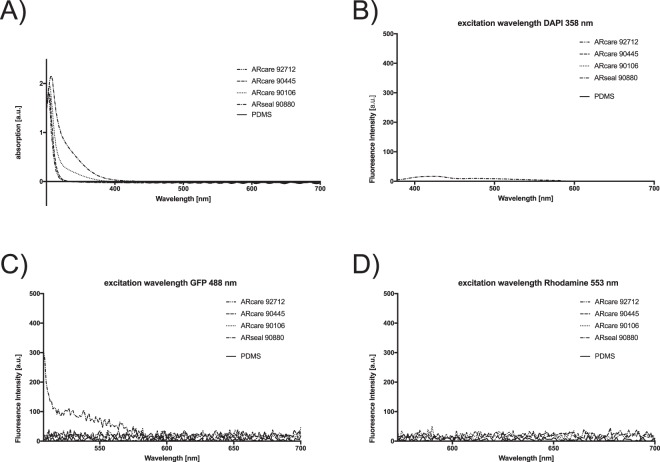
Optical characterization of ARcare and ARseal pressure sensitive adhesives and PDMS for (**A**) Absorbance spectra and (**C**,**D**) autofluorescence spectra at three commonly used excitation wavelengths for fluorophores applied to cell-based assays. Data points are presented as mean values for n = 4. For mean values ± SD see SI Fig. 3.

Another important material characteristic of a microfluidic cell culture device is their optical transparency and autofluorescence, since most cell-based assays to date are based on imaging methods. Consequently, optical characterization of pressure sensitive adhesives included absorbance spectra and autofluorescence spectra using three excitation wavelengths of commonly employed fluorophores in cell-based assays. Figure 6A demonstrate similar transparencies for all ARcare adhesives and the ARseal pressure sensitive adhesive with no detectable absorbance above 400 nm wavelength (ARcare 92712 and ARcare 90445 even above 320 nm). Additionally, Fig. 6B–D verifies the absence of autofluorescence in the presence of the ARcare type adhesives at any excitation wavelengths, while low autofluorescence was observed for ARseal 90880 at an excitation wavelength of 358 nm below an emission wavelength of 450 nm as well as excitation at 488 nm below an emission wavelength of 575 nm. PDMS as a reference showed no optical interferences for absorption as well as excitations fir 358 nm, 488 nm and 553 nm. These results clearly demonstrate that all selected pressure sensitive biomedical adhesives are well suited for cell-based biochip assays based on optical read-outs.

### Practical applications of pressure-sensitive adhesives in rapid prototyping of cell-based lab-on-a-chip and organ-on-a-chip systems

**Figure 7:**
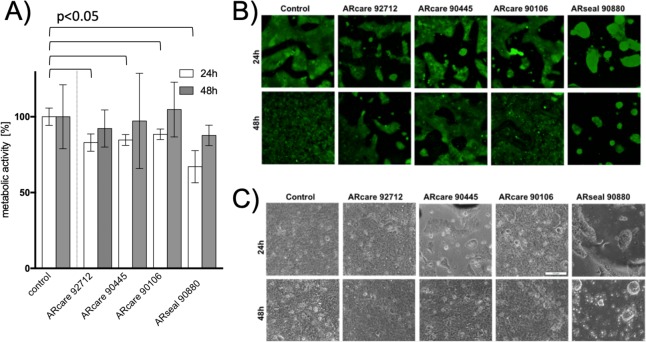
Biocompatibility of biomedical-grade pressure sensitive adhesives including (**A**) metabolic activity Data bars are mean values ± SD for n = 3 (**B**) viability and (**C**) adhesion of BeWo b30 epithelial cells. Viability is expressed as percentage of living cells normalized to control glass substrates after 24 and 48 h post-seeding.

Prior to application of medical-grade pressure sensitive adhesives in rapid prototyping for cell-based microfluidic assays, biocompatibilities were investigated using metabolic as well as live/dead bioassays to rule out any detrimental effects of the chemical adhesive layers on living cells. Figure 7A shows obtained cell viabilities of BeWo placental epithelial cells cultivated directly on the adhesive layer of the tapes to promote maximum exposure to chemical leachable substances. BeWo cells growing on top of ARcare type adhesives (e.g. 90445, 90106, and 92712) showed superior metabolic activity >85% in comparison to bare glass surfaces following a 48 h incubation period (p > 0.58). Additionally, the increased metabolic activities in the presence of adhesives indicates normal and healthy cell growth. In contrast, epithelial cells grown on top of ARseal 90880 displayed reduced metabolic activities of 67 ± 7% and 88 ± 7%, respectively. Furthermore, live/dead dye-exclusion assays based on calcein AM and quantitation of necrotic cells by ethidium bromide staining results showed similar degree of cell death for all medical-grade adhesive tapes (see Fig. 7B). The observed metabolic variations between the different adhesive tapes point at cell adhesion phenomena known to influence cell metabolism. For instance, as shown in Fig. 7C, BeWo epithelial cells are able to attach and form confluent monolayers on acrylic adhesive of all ARcare types, while the silicone-based adhesive of ARseal showed cell repellant properties promoting the formation of epithelial cell spheroids.

**Figure 8:**
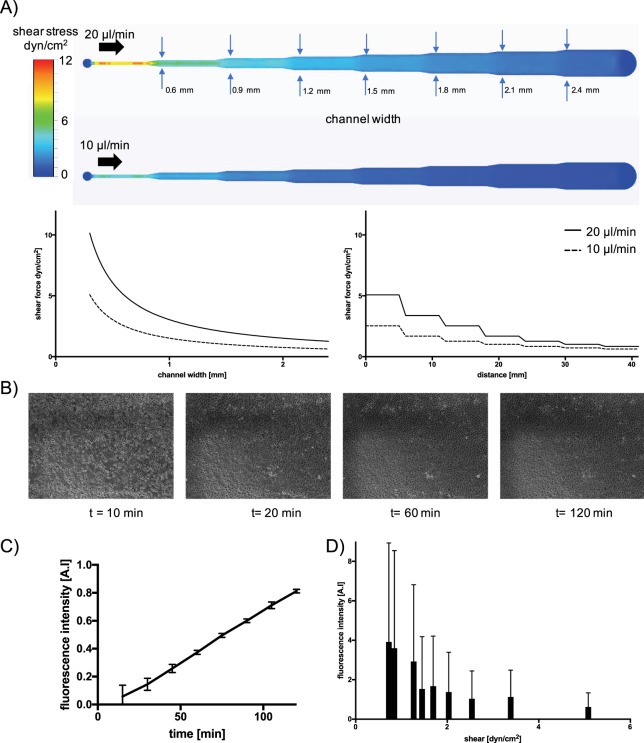
(**A**) CFD simulation of shear stress within a PSA rapid prototyped microfluidic biochip perfused with either 20 µl/min (top) and 10 µl/min flow (bottom), and simulated shear-force over distance and channel width (bottom panels). (**B**) Time-lapse microscopy of HUVEC endothelial cell adhesion starting 10 minutes post-seeding. (**C,D**) Impact of exposure time (**C**) and shear (**D**) on uptake of non-toxic fluorescent polystyrene 250 nm nanoparticles on confluent HUVEC endothelial cells. Data points are presented as mean values ± SD for n = 4.

Another practical demonstration of using medical-grade adhesives for rapid prototyping of cell-based microfluidic assays involves shear-dependent uptake of non-toxic fluorescently labelled nanoparticles on human endothelial cells. Here, a multi-channel biochip was fabricated using ARcare 90445 featuring declining channel width to subject endothelial cells to increasing shear stress in the range of 0–5 dyn/cm^2^. To estimate obtained shear forces along a decreasing microchannel width a set of CFD simulations were performed prior experimentation. Figure 8A shows calculated fluid shear values in the presence of 10 µl/min and 20 µl/min flow rates, where an increase of cross-sectional area from 0.6 mm to 2.4 mm reduced fluid shear stress from 2.54 to 0.63 dyn/cm^2^ and 5.08 to 1.27 dyn/cm^2^, respectively. To ensure similar endothelial cell coverage along the decreasing collagen I-coated microchannels sections, HUVEC endothelial cells adhesion on biochip interfaces was monitored over a period of 2 hours using live-cell microscopy. Images shown in Fig. 8B indicated similar HUVEC cell attachment behavior within <20 min after cell seeding, followed by evenly spreading throughout the entire microchannel geometry over the next 100 minutes, thus indicating the formation of a homogeneous cell barrier. To ensure proper barrier integrity, on-chip cultivation of confluent HUVEC endothelial cells was allowed to take place over a period of 2 days prior treatment with medium supplemented with 4% fluorescently-labelled 249 nm-sized polystyrene nanoparticles. Results of our shear-dependent nanoparticle uptake study are shown in Fig. 8C,D, where HUVEC were exposed to nanoparticles over period of 2 hours at 10 µl/min and 20 µl/min flow rates. This means that in our experimental design configuration shear forces in the range of 0.5 to 5 dyn/cm^2^ can be investigated to study the impact of flow conditions on cellular nanoparticle uptake rates. As an example, Fig. 8C shows a linear nanoparticle uptake rate over a 2 hour period by HUVEC in the presence of 0.7 dyn/cm^2^ (e.g. 10 µl/min flow rate using a 2.1 mm width section), thus pointing at a constant and stable cellular nanoparticle uptake behavior. Notably, a 14-fold fluorescence signal increase is already obtained over a 100 min period with good read-out sensitivity and reproducibility of 0.06 ± 0.07 to 0.82 ± 0.01. In a final set of experiments, shear-dependent cellular uptake behavior following a 2 hour exposure duration was investigated to assess the influence of flow velocity on nanoparticle uptake capacity. As seen in Fig. 8D a signal decrease from 3.9 ± 5 at 0.7 dyn/cm^2^ to 1.0 ± 1.4 at 2.5 dyn/cm^2^ can be observed indicating a reduced capacity of HUVEC to actively take up 249 nm nanoparticles.

## Discussion

It is well known that both the bio-interface and applied flow condition influence cell behavior within microfluidic devices including metabolic activity, cell attachment, differentiation, barrier formation and nanoparticle uptake. Consequently, knowledge of material properties such as biocompatibility and applied shear force conditions play a vital role when developing cell-based lab-on-a-chip and organ-on-a-chip systems. In this work, we have characterized four pressure sensitive biomedical adhesives for rapid prototyping of cell-based lab-on-a-chip and organ-on-a-chip microsystems with the aim of providing detailed information on their advantages and limitations. A summary of our characterization results is listed in Table 2, showing excellent optical properties, good manufacturability, low height tolerances and strong bonding to both glass and a variety of polymeric materials such as membranes. The ability to rapidly assemble multi-layered and membrane-integrated microfluidic devices is of great interest when studying cell barrier integrity and mechanobiological stimuli. Additionally, reliable and stable biochip operation can be maintained over several weeks in the presence of high flow rates, physiological temperatures (e.g. 37 °C) and 100% humidity. While ARcare 90106 adhesive displayed gas permeability (p < 0.001), which is crucial for long-term cell culture conditions, ARcare 90445 showed good combination of properties in regards of bonding strength to various materials, cutting tolerance, cell adhesion and viability as well as gas tightness. Furthermore, controlling bonding pressure during device assembly is crucial in order to reduce variations in channel height between individual devices, while the application of bonding pressures above 2 kN eliminates unwanted swelling effects during prolonged cell culture. Height variations lead to altered fluid dynamics inside microchannels that result in unpredictable shear forces, which ultimately lead to non-reproducible measurement conditions in shear-dependent cell studies.

**Table 2:** Comparison between ARcare and ARseal biomedical-grade pressure sensitive adhesive tapes.

Pressure Sensitive Adhesive	ARcare 92712	ARcare 90445	ARcare 90106	ARseal 90880
% max. tolerance (channel size in µm)	16.6 (@250)	19.9 (@200)	10.1 (@350)	17.1 (@200)
% channel height tolerance by manual bonding/2 kN bonding pressure	5.2/5.6	16.7/2.4	36.1/7.5	8.0/5.7
tensile strength glass incubated at 37 °C [MPa]	0.7	0.76	0.41	0.78
tensile strength PET membrane at 37 °C [MPa]	0.48	0.72	0	0.68
shear force glass [MPa]	1.44	3.23	0.77	0.77
shear force PET membrane [MPa]	2.34	2.79	0.51	0.56
oxygen permeability (ranked 1 best 4 worst)	3	2	1	4
vapor permeability (ranked 1 best 4 worst)	4	2	1	3
cell adhesion	+	+	+	−
cell metabolism	+	+	+	~
cell viability	+	+	+	+

Our biological characterization demonstrated excellent biocompatibilities of all pressure-sensitive tapes with the limitation of ARseal 90880, which led to the formation of cell aggregates pointing at low adhesive properties of the silicone-based adhesive. Our final practical demonstration of using the medical-grade adhesives ARcare 90106 for rapid prototyping of cell-based microfluidic assays investigate the time- and shear-dependent uptake of non-toxic fluorescently labelled nanoparticles by human endothelial cells. Using a tapered microfluidic channel layout linear uptake of nanoparticles by HUVECS over a period of 2 hours was found, while a progressive increase of shear force conditions resulted in reduced uptake capacity of 240 nm sized nanoparticles. Interestingly above the threshold of 2.5 dyn/cm^2^ nanoparticle uptake becomes independent from the applied shear force, which represents a broad range of physiological shear forces present at starts at the postcapillary venules (e.g. 1–5 dyn/cm^−2^) and arteries (e.g. 20–30 dyn/cm^−2^)^41^.

Overall, rapid prototyping using pressure sensitive adhesive tapes allows for one-step manufacturing with fast concept-to-chip time and its application is highly feasible even for cell-based microfluidic devices that require multiple stacked layers as well as integrated porous membranes. We believe that medical-grade pressure sensitive adhesive tapes present a viable alternative to overcome the challenge of integrating multiple functional layers of different polymer types including rigid pneumatic and fluidic layers as well as flexible membranes in a fast and reproducible manner.

## Material and Methods

### Micro machining of pressure sensitive adhesive films using plotting technology

High performance biomedical pressure sensitive adhesives (PSA) were chosen for this work. The adhesives were utilized in four configurations: (1) ARcare 92712® (Adhesive Research, UK), a clear pressure sensitive double-sided adhesive tape containing a 12.7 μm thick polyester film with MA-93 as acrylic pressure sensitive adhesive (17.78 μm on each side) with a total thickness of 48.26 μm (with liners 149.86 μm); (2) ARcare 90445^®^ (Adhesive Research, UK) is a clear pressure sensitive double-sided adhesive tape out of a 25.4 μm thick polyester film with AS-110 acrylic medical grade adhesive (27.94 μm on each side) with results in a total thickness of 81.28 μm (with liners 182.88 μm); (3) ARcare 90106^®^ (Adhesive Research, UK) is a clear pressure sensitive double-sided adhesive tape out of a 25.4 μm thick polyester film with MA-69 acrylic hybrid medical grade adhesive (58.42 μm on each side) with results in a total thickness of 142.24 μm (with liners 243.84 μm) and (4) ARseal 90880^®^ (Adhesive Research, UK) is a clear pressure sensitive double-sided adhesive tape out of a 50.8 μm thick polypropylene film with SR-26 silicone adhesive (45.72 μm on each side) with results in a total thickness of 142.24 μm (with liners 243.84 μm). All shapes in the pressure sensitive double-sided adhesive tapes and PDMS foil (Super clear 0.25 mm MVQ Silicones GmbH) were designed using with AutoCAD 2017 (Autodesk) and copied into CutStudio (Roland). Plotting was performed by a CAMM-1 Servo GX-25 (Roland) with a ZEC-U5032 (Roland) blade. The pictures of the cutting quality were analyzed using FIJI software (ImageJ, USA). Inlet holes were drilled manual in glass slides (VWR, Austria). Bonding of the pressure sensitive adhesive tape and bottom and top glass layers was performed by applying pressure of 2 kN for 1 minute. Bonding of PDMS foil was performed by plasma activation (high) for 2 min (Blackhole lab plasma cleaner) and curing for 10 min at 80 °C for given pressure.

### Evaluation of optical properties

Pressure sensitive adhesives samples bonded between two clean glass slides with 2 kN for 1 min and PDMS samples were prepared as described before. For quantification of absorption and auto-fluorescence an EnSpire 2300 plate reader (PerkinElmer) was used. Values were normalized to glass as a base line value. The fluorescence excitation and corresponding emission spectra were measured for wavelengths of 358 nm/378–700 nm, 488 nm/508–700 nm and 553 nm/573–700 nm, whereas absorbance was scanned at wavelength from 300 nm to 700 nm.

### Oxygen permeability

Oxygen monitoring was carried out at a sampling frequency of 1 Hz using a FireStingO2 optical oxygen meter (Pyroscience GmbH, Germany) connected to optical fibers (Circular structure with an inner diameter of 4 mm and an outer diameter 6 mm, 1 mm wall thickness). Integrated sensors were calibrated using a CO_2_/O_2_ oxygen controller (Pecon GmbH, Germany) equipped with integrated zirconium oxide oxygen sensors. Oxygen measurements were initiated directly after manual bonding of the chip and partial oxygen pressure was monitored up to a maximum duration of 22 hours at 37 °C, 5% CO_2_ and 0–21% O_2_.

### Vapor permeability

Microchannels of 2 mm × 20 mm in size with a wall thickness of 3 mm were manual bonded between glass slides with powder-blasted holes, filled with water and sealed with PCR foil (Sarstedt, Austria). The samples were heated at 37 °C for 24 h. The size of air bubbles was measured every hour using CellSens software (Olympus, Germany). The relative bubble growth was calculated by dividing every measurement by the initial value.

### Tensile and shear strength test

For determining the tensile and shear strength of each PSA type a circular area of 1 cm^2^ was bonded between two glass slides with 250 N applied pressure for 1 min in a shop press WP 20 H (Holzmann Maschinen, Austria) equipped with a precision tension and compression load cell 8524 (Burster, Germany). For testing the strength of bonding to membranes the same procedure was conducted by placing a membrane between two 1 cm^2^ PSA layers laminated on glass slides. Force was applied by a rate of approximately 10 N/s. After each cycle, delamination of the samples from the PSA checked and samples delaminated from glass were excluded from the membrane experiments.

### CFD simulation

The CFD simulation was performed by CFD Autodesk 2019. The CAD model of the chip was created in Fusion 360 (Autodesk). The bottom and the top layer of the chip has been modeled as glass and the pressure sensitive adhesive with the given channel structure as acrylic. The fluid was modeled as water at room temperature. Furthermore, there was no heat exchange and gravity simulated. The fluid inlets were modeled by a defined volume flow by 10 mm^3^/h respectively 20 mm^3^/h. The outlets were modeled as openings with 0 pascal pressure. No further initial conditions were added, and the net was generated automatically by the software.

### Cell culture

Human umbilical vein endothelial cells (HUVEC, C2519A, Lonza) were maintained in Endoprime base medium supplemented with 5% fetal calf serum, 5% human serum, 0.2% VEGF and 0.2% EGF (Endoprime supplement kit; PAA laboratories GmbH) at 37 °C and humidified atmosphere. BeWo b30 cells (kindly provided by Dr. Tina Buerki-Thurnherr, EMPA, Switzerland) were cultivated in Dulbecco’s modified Eagle medium supplemented with 10% FCS, L-glutamine and high glucose (Sigma Aldrich).

### Biocompatibility

To evaluate the biocompatibility, circular (13 mm diameter) PSA sheets were bonded on the bottom of a well in 24 well plate and protective liners were removed. Cells were directly seeded onto the adhesive layers at an initial seeding density of 10^5^/cm^2^. Pictures were taken 24 h and 48 h after seeding. For live/dead staining, Calcein-AM and Ethidium Homodimer-1 (Thermo Fisher Scientific, Austria) was used as described in the manufacturer’s description and incubated for 30 min at 37 °C. To quantify viability via metabolic activity, a 10% Presto blue reaction mix diluted in complete cell culture media (Thermo Fisher Scientific, Austria) was incubated for 30 min and quantified using an EnSpire 2300 plate reader (PerkinElmer, Austria) with Presto blue reaction mix as baseline. Cell samples without any PSA material was taken as reference for a viability of 100%. T-test was performed to evaluated significance between the control values and the values for each tape after 24 h receptivity 48 h. For p values see SI Table 1.

### Microfluidic cell-culture & nanoparticle uptake

For cell culturing the chip was disinfected with 70% ethanol for 30 min and flushed twice with phosphate buffered saline (PBS, Sigma-Aldrich, Austria) before priming with supplemented endothelial growth medium. For surface-modification, the chips were treated with Collagen Type I from rat tail (0.1% solution in DPBS, Sigma-Aldrich, Austria) for 30 min and flushed with culture medium. For initiation of culture, a T75 roux flask of HUVECs was pelleted and cells were introduced at 100% confluency corresponding to an initial seeding density of 3*10^4^ cells/cm^2^ and allowed to attach. After 10 minutes perfusion culture was started at a flow rate of 1 µl/min using a KDS scientific four channel syringe pump (KDS Scientific). For shear experiments, the initial flow rate for perfusion culture was increased to 10–20 µl/min.

For nanoparticle uptake studies, non-toxic yellow fluorescent particles with a mean diameter of 249 nm (PFP-0252; Kisker Biotech) were sonicated for 5 minutes and supplemented with complete culture medium to a final working concentration of 4% (40 µL of the NP stock in 960 µL culture medium resulting in 0.4 mg/ml). Following adjustment of the HUVEC cells within the microchannels for 1–2 days, nanoparticle exposure was initiated for 6 hours using the shear chips at either 10 or 20 µl/min to generate two different shear regimes. After 6 hours of imaging using an IX83 live-cell microscope with temperature and CO_2_ control (Olympus, Austria), particle uptake was analyzed from time-lapse imaging every 10 minutes using FIJI software (ImageJ, USA). A minimum of 10 cells from the middle of the channel in each condition were analyzed and fluorescence intensity of the cell area was averaged using Origin 8 Pro software for data analysis.

## Supplementary information

Supporting information - Characterization of four functional biocompatible pressure-sensitive adhesives for rapid prototyping of cell-based lab-on-a-chip and organ-on-a-chip systems
