# Supplementary material for: Characterization of four functional biocompatible pressure-sensitive adhesives for rapid prototyping of cell-based lab-on-a-chip and organ-on-a-chip systems
Source: Sci Rep. 2019 Jun 26;9:9287. doi: 10.1038/s41598-019-45633-x (PMC6594959; doi:10.1038/s41598-019-45633-x)
Supplement: Supplementary file 1 — Supporting information - Characterization of four functional biocompatible pressure-sensitive adhesives for rapid prototyping of cell-based lab-on-a-chip and organ-on-a-chip systems [file 41598_2019_45633_MOESM1_ESM.pdf]

Supporting information - Characterization of four functional biocompatible pressure-sensitive adhesives for rapid prototyping of cell-based lab-on-a-chip and organ-on-a-chip systems

S.R.A. Kratz [1,2,ˀ], C. Eilenberger [1,2,ˀ], P. Schuller [1,2],B. Bachmann [1,2,3], S. Spitz [1,2], P. Ertl [1,2*] and M. Rothbauer[1,2,ˀ,*]

[1] *Institute of Applied Synthetic Chemistry and Institute of Chemical Technologies and Analytics, Faculty of Technical Chemistry, Vienna University of Technology, Vienna, Getreidemarkt 9/163-164, 1060 Vienna, Austria*

[2] *Austrian Cluster for Tissue Regeneration, Vienna, Austria*

[3] *Ludwig Boltzmann Institute for Experimental and Clinical Traumatology, Allgemeine Unfallversicherungsanstalt (AUVA) Research Centre, Vienna, Austria, Donaueschingenstraße 13, 1200 Vienna, Austria*

* peter.ertl@tuwien.ac.at

* mario.rothbauer@tuwien.ac.at

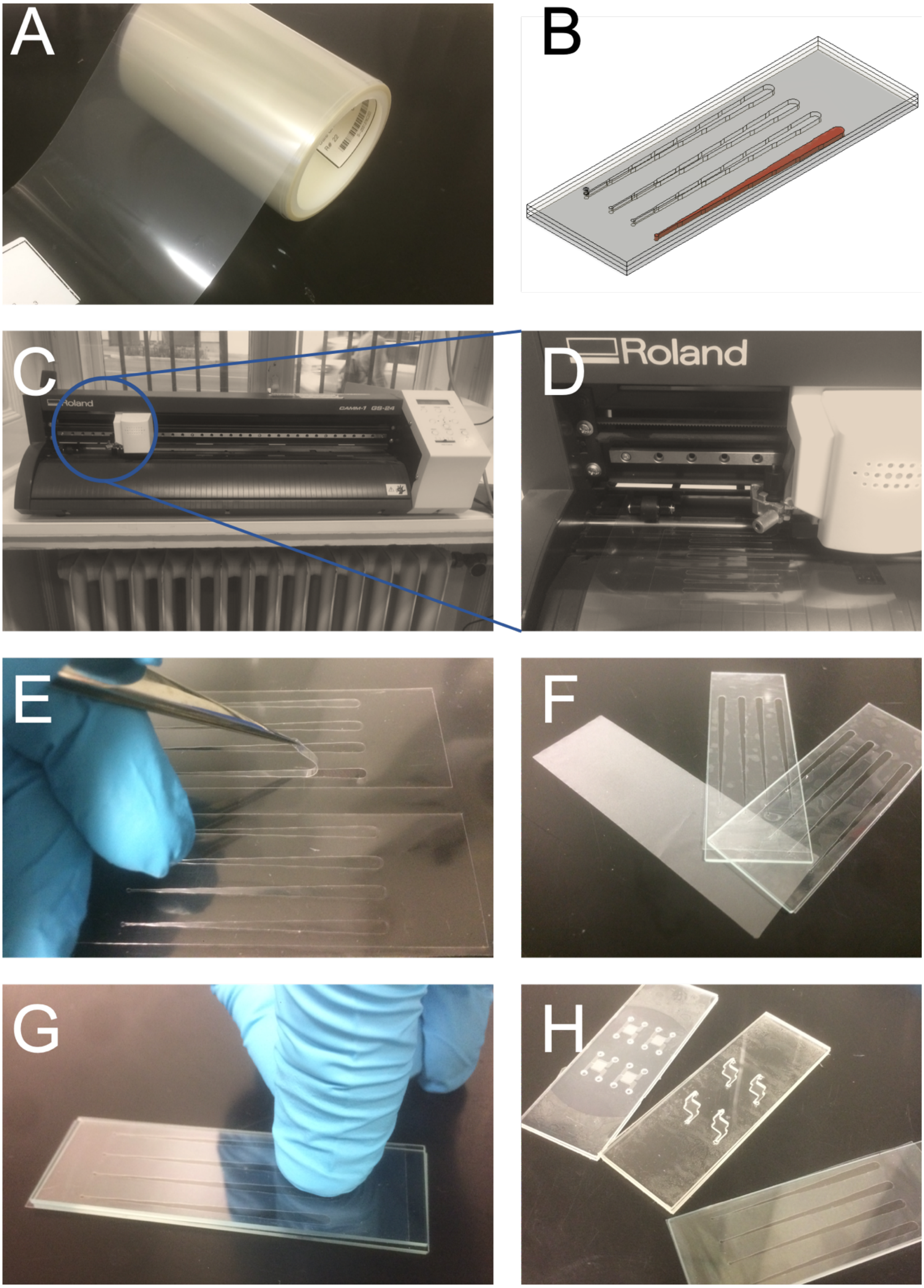

SI- Fig 1 A) Pressure sensitive adhesive (PSA) B) CAD model of microfluidic device C) desktop vinyl cutter D) PSA loaded in vinyl cutter with cut structure E) Peeling of residual structures F) Parts to assemble (PSA, glass slides, membranes) G) Device bonding by applying pressure H) Assembled devices

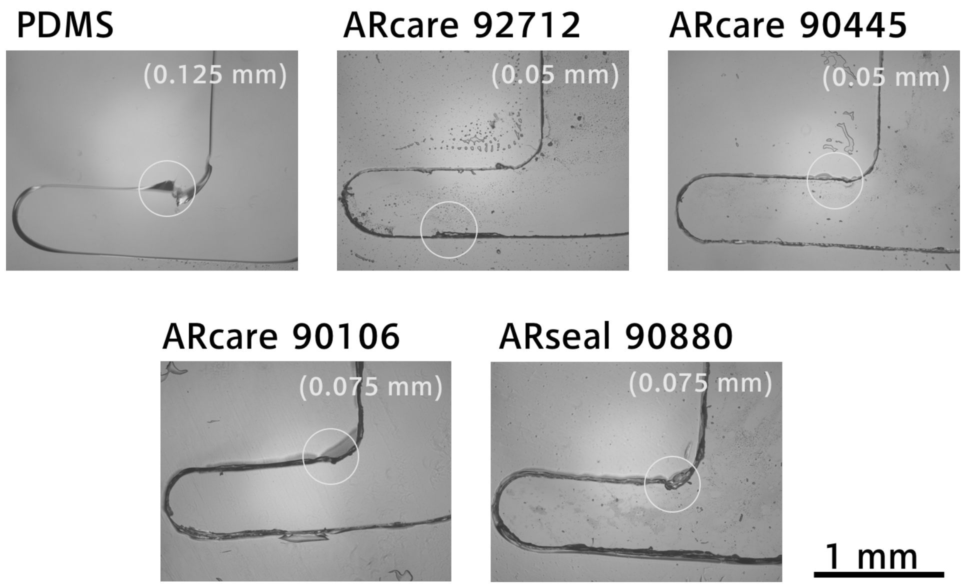

SI- Fig 2 Cutting behavior of PDMS (250 µm thick), ARcare 92712, ARcare 90445, ARcare 90106 and ARseal 90880. The circles are marking the region with the most deformation of the designed structures. The number in brackets indicate the scale of the deformation.

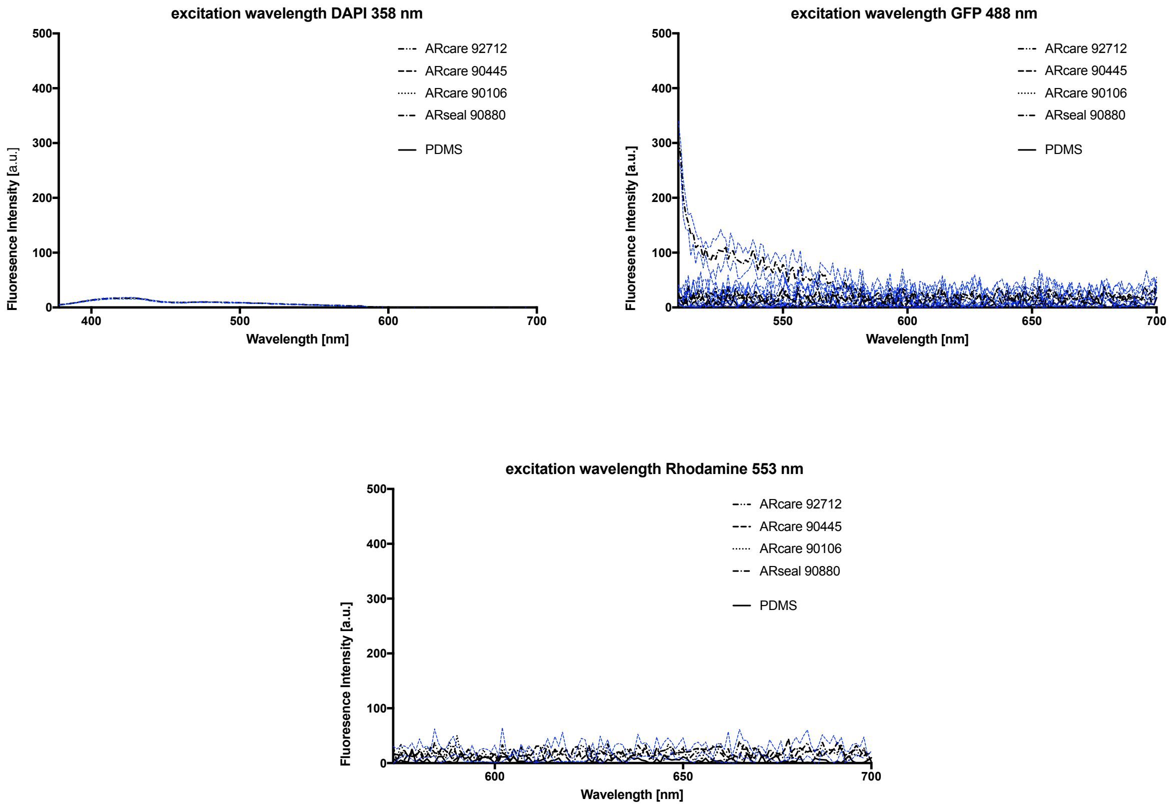

SI- Fig 3 Optical characterization of ARcare and ARseal pressure sensitive adhesives and PDMS for (A) Absorbance spectra and (C-D) autofluorescence spectra at three commonly used excitation wavelengths for fluorophores applied to cell-based assays. Data points are presented as mean values ± SD (blue) for n = 4.

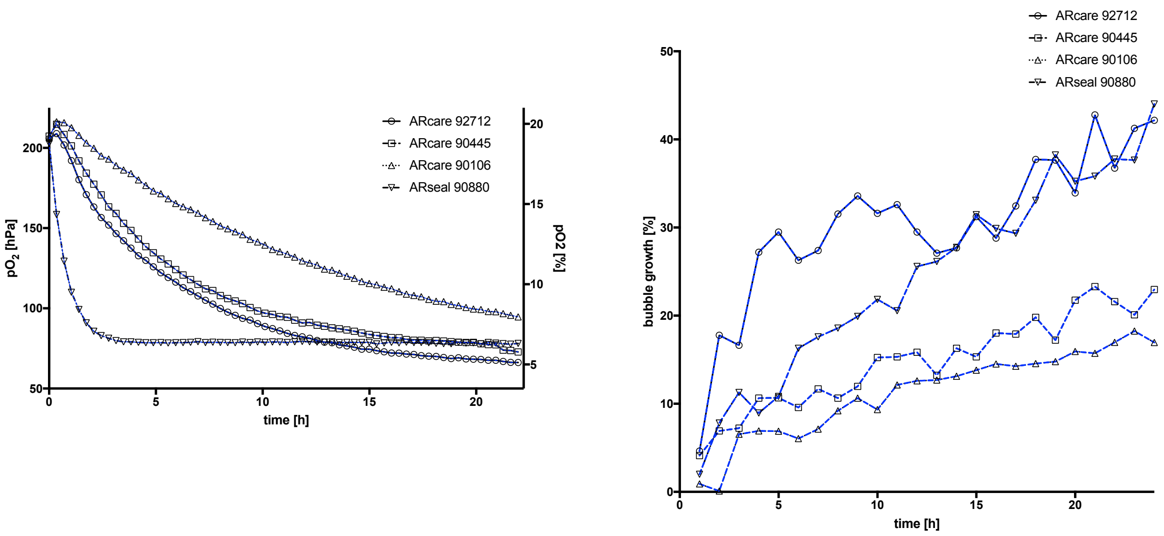

SI- Fig 4 Oxygen: where in a circular structure with a wall thickness of 1mm (blue units in mm) oxygen is measured by micro sensor (green) and (B) vapor permeability of biomedical pressure sensitive adhesives: where medium (red) evaporation is measured through rectangular structure with a wall thickness of 3 mm. Data points are presented as mean values ± SD (blue) for n = 4.

SI – Tab 1. p-values for viability assay of PSA

24h	48h	Adhesive
p= 0.0039	p= 0.58	ARcare 92712
p= 0.0041	p= 0.877	ARcare 90445
p= 0.0158	p= 0.75	ARcare 90106
p= 0.0004	p= 0.37	ARseal 90880
